# 골다공증이 있는 폐경 후 당뇨 여성의 건강관련 삶의 질 영향요인: 제7기 국민건강영양조사 자료(2016–2018년) 활용

**DOI:** 10.4069/kjwhn.2022.05.25

**Published:** 2022-06-29

**Authors:** Hyuk Joon Kim, Hye Young Kim

**Affiliations:** College of Nursing, Jeonbuk National University, Jeonju, Korea; 전북대학교 간호대학

**Keywords:** Diabetes mellitus, Osteoporosis, Postmenopause, Quality of life, Women, 당뇨병, 골다공증, 폐경, 삶의 질, 여성

## Abstract

**Purpose:**

The prevalence of osteoporosis in postmenopausal women is increasing, and diabetes patients have decreased bone density. Their health-related quality of life (HRQoL) is diminished by the resultant physical dysfunction and depression. The purpose of this study was to identify factors influencing HRQoL in postmenopausal women with diabetes and osteoporosis.

**Methods:**

This was a secondary data analysis of the Seventh Korea Health and Nutrition Examination Survey (2016–2018), which utilized a complex, multistage probability sample design. The participants in the study were 237 women with diabetes and osteoporosis. To evaluate the factors that influenced HRQoL, a complex-samples general linear model was constructed, and the Bonferroni correction was performed.

**Results:**

In this sample of women aged 45 to 80 years (mean±standard deviation, 71.12±7.21 years), the average HRQoL score was 0.83±0.18 out of 1.0. Factors influencing HRQoL were age (70s: t=–3.74, *p*<.001; 80s: t=–3.42, *p*=.001), walking for exercise more than 5 days a week (t=–2.83, *p*=.005), cerebrovascular disease (t=–8.33, *p*<.001), osteoarthritis (t=–2.04, *p*=.014), hypertension (t=2.03, *p*=.044), higher perceived stress (t=–2.17, *p*=.032), poor glycemic control (t=3.40, *p*=.001), waist circumference (t=–2.76, *p*=.007), sitting time per day (t=–2.10, *p*=.038), and a longer postmenopausal period (t=3.09, *p*=.002).

**Conclusion:**

In order to improve the HRQoL of postmenopausal women with osteoporosis and diabetes, it is necessary to implement intervention strategies that enable the effective management of chronic diseases, while preventing the complications of diabetes and minimizing stress through physical activity.

## Introduction

골다공증은 중년기 여성의 삶의 질을 저하시키는 질환 중 하나[1]로 2018년 국민건강영양조사 자료에 의하면 50세 이상 중 골다공증으로 진단받은 비율은 남성(1.8%)보다 여성(24%)에서 더 높고, 특히 폐경 후 여성에서는 26.1%를 차지했다[2]. 골다공증은 골밀도의 감소로 골 강도가 약해지면서 가벼운 충격에도 골절이 쉽게 발생하는 질환이다[1]. 여성은 남성보다 골 생성량이 적고 폐경으로 인한 에스트로겐의 감소로 골 소실이 급격하게 발생하기 때문에 골다공증의 발병률이 남성보다 높다[3]. 골다공증으로 인한 골절은 통증, 기형, 장애 등을 초래할 뿐만 아니라 골절을 경험한 여성에게 의료비 부담의 증가, 사회활동의 제한, 우울 등 심리•사회적으로 부정적인 영향을 미쳐 궁극적으로 이들의 삶의 질을 저하시킨다[1]. 여성에게 있어 폐경 후 삶이 전체 생애의 1/3 이상임을 고려할 때 인구 고령화와 함께 폐경 후 여성의 건강관련 삶의 질에 관심을 가질 필요가 있다.

폐경 후 여성에게서 당뇨병은 골다공증과 밀접한 관련이 있다[4]. 여러 역학 연구[5,6]에서 골다공증과 당뇨병에 대한 관련성을 보고하였고, 메타분석 연구[4]에서도 당뇨병 환자는 당뇨병이 없는 여성에 비해 골다공증과 함께 고관절 골절이 증가하는 것으로 나타났다. 당뇨병 환자의 경우 당화혈색소가 높아지면 골밀도가 감소하고, 부갑상선 호르몬의 증가는 골 소실을 유발하여 골다공증을 초래한다[7]. 또한 당뇨병 치료제 중 일부는 골 대사에 영향을 주어 상‧하지 골절의 위험을 증가시킨다[7]. 2019년 국내 30세 이상 여성의 당뇨병 유병률은 12.7%로 2011년 10.4%에서 꾸준히 증가하고 있어[8], 당뇨병은 골다공증과 함께 폐경 후 여성의 건강을 위협하는 심각한 문제로 여겨진다.

당뇨병은 완치할 수 없는 질환으로 당뇨병 환자는 엄격한 생활 습관과 함께 평생 혈당을 조절해야 한다는 부담감, 당뇨병으로 인한 합병증에 대한 두려움, 잦은 입‧퇴원, 치료에 대한 경제적 부담 등으로 다른 만성질환자보다 대상자가 지각하는 삶의 질의 수준이 낮다[5]. 특히 당뇨병성 합병증을 가진 환자에게 골절이 동반되면 골절의 치유가 지연되고 삶의 질은 더욱더 위협받게 된다[6]. 일반적으로 낮은 수준의 삶의 질은 절망감, 우울에 이어 자살 생각을 유발하는 주요한 변수로 작용할 수 있기에[9] 골다공증과 당뇨병을 동반한 폐경 후 여성의 삶의 질에 관심을 가질 필요가 있다.

선행연구에 따르면 폐경 후 여성에서의 골다공증 유병률은 폐경기간이 길어질수록 높아지고[10], 이들의 삶의 질은 동반하는 만성질환 수가 많을수록, 우울 수준이 높을수록[11], 교육 수준이 낮거나, 스트레스 수준이 높을 때, 배우자나 직업이 없을 때 감소하는 것으로 나타나[6] 폐경 후에서 노년기로 이어지면서 만성질환의 발생이나 우울과 스트레스 수준 등이 삶의 질과 관련이 있는 것으로 나타났다. 또한 당뇨병 환자의 삶의 질은 여성이 남성보다, 연령이 많을수록, 당뇨병성 합병증이 있거나 당뇨병 유병기간이 길수록 더 낮은 것으로 보고되었다[5]. 특히 폐경 후 당뇨 여성에게 골다공증의 동반은 골절로 인한 활동 장애, 통증[11]과 함께 낙상이나 골절로 인한 심각한 장애를 유발하여 이들의 사망률을 높인다[12]. 당뇨병이나 폐경에 동반되는 만성질환 등에 대한 질병 관리의 부담, 불안, 우울 등은 이들의 삶의 질을 감소시킨다[11,13]. 따라서 폐경 후 여성의 삶의 질은 신체적, 심리적, 정서적 건강에 직접적인 영향을 미치고 더 나아가 성공적인 노후로 연결되므로, 폐경 후 여성의 삶의 질과 관련된 요인들을 확인하고 이를 중재하는 것은 의미 있는 작업이다.

폐경 후 여성을 대상으로 한 선행연구의 대부분[5,10,12]은 편의표본을 이용하여 골다공증 또는 당뇨병과 삶의 질 간의 관계를 밝히거나 관련 요인을 단변량적 분석을 통해 보고하였다. 이로 인해 삶의 질에 영향을 미치는 요인들을 통합적으로 파악하지 못하거나 결과의 일반화에 제한점을 가지고 있다. 이에 본 연구는 국민건강영양조사 제7기 자료를 이용하여 골다공증이 있는 폐경 후 당뇨 여성의 삶의 질 영향요인을 다변량 분석법을 통해 통합적으로 평가함으로써 조절 가능한 요인들을 관리하여 골다공증이 있는 폐경 이후 여성의 삶의 질을 향상하는 데 기여하고자 한다.

본 연구는 골다공증이 있는 폐경 후 당뇨 여성의 건강관련 삶의 질에 영향을 미치는 요인을 확인하기 위하여 시행되었으며, 구체적인 목적은 다음과 같다. (1) 대상자의 인구사회학적 특성, 생활습관 관련 특성에 따른 건강관련 삶의 질의 차이를 파악한다. (2) 대상자의 건강관련 특성에 따른 건강관련 삶의 질의 차이를 파악한다. (3) 대상자의 건강관련 삶의 질에 영향을 미치는 관련 요인을 확인한다.

## Methods

Ethics statement: Obtaining informed consent was exempted by the Institutional Review Board of Jeonbuk National University (JBNU 2022-01-019-001) as this study was secondary analysis of existing data and used anonymized data.

### 연구 설계

본 연구는 국민건강영양조사 제7기(2016–2018년)의 자료[1]를 이용하여 골다공증이 있는 폐경 후 당뇨 여성의 건강관련 삶의 질에 영향을 미치는 요인을 파악하기 위한 이차자료 분석 연구이다. 연구의 기술은 STROBE 보고지침(https://www.strobe-statement.org/)에 따라 작성하였다.

### 연구 대상

**Figure 1.**
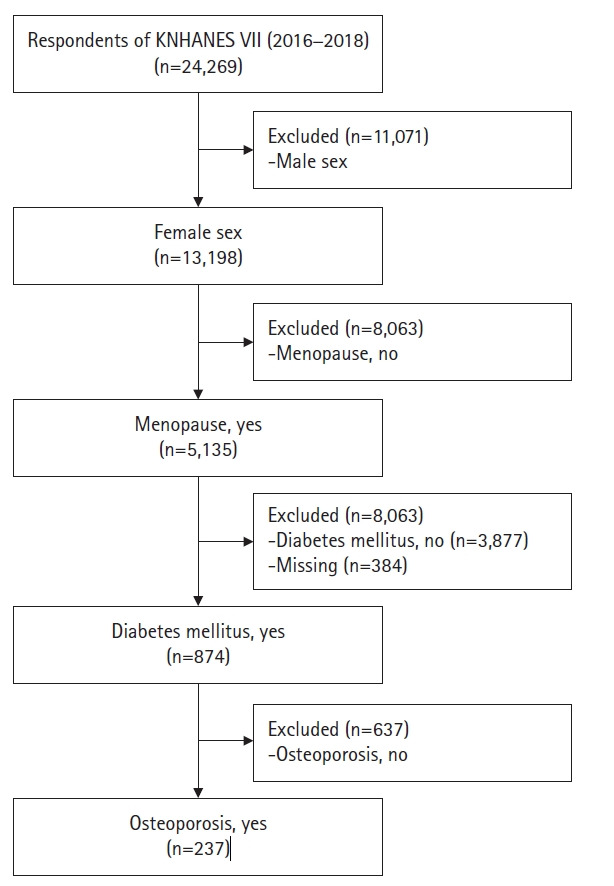
Flow chart showing the selection of the study participants KNHANES: Korea National Health and Nutrition Examination Survey.

본 연구의 대상은 국민건강영양조사의 원시자료 중 골다공증에 대한 조사가 시행된 2016–2018년 자료이다. 우리나라 전 국민을 대상으로 층화표본 추출방법에 의하여 2016–2018년에 실시된 국민건강영양조사 대상자 24,269명 중 일차로 여성 13,198명을 추출하였고, 그중 폐경을 경험한 여성 5,135명을 추출하였다. 여기에서 다시 당뇨로 진단받은 874명을, 그리고 마지막으로 골다공증이 있는 237명을 추출하여 본 연구의 최종 분석 대상자는 총 237명이었다 (Figure 1).

### 연구 도구

본 연구의 종속변수는 건강관련 삶의 질이고, 독립변수는 선행연구에서 보고된 폐경 후 골다공증 또는 당뇨병 환자의 삶의 질에 영향을 주는 변수 중 국민건강영양조사 자료에서 활용 가능한 변수들을 추출하여 사용하였다. 독립변수는 일반적 특성 및 생활습관, 건강관련 특성으로 구분하였으며, 각 변수의 범주는 국민건강영양조사의 범주를 그대로 사용하거나 본 연구자들이 재분류하여 사용하였다.

#### 건강관련 삶의 질

대상자의 건강관련 삶의 질은 EuroQol Group이 개발한 EQ-5D (EuroQol-5 dimension)를 이용하여 측정한 값을 분석하였다. EQ-5D는 건강관련 삶의 질을 측정하는 도구로서 전반적인 건강을 측정하기 위해 개발되었으며, 운동능력, 자기관리, 일상활동, 통증/불편, 불안/우울의 5개의 객관식 문항으로 구성되어 있다. 각 문항의 측정값에 대하여 가중치를 적용하여 건강관련 삶의 질 점수인 EQ-5D index를 구하게 된다. 본 연구에서는 국민건강영양조사에서 질병관리청의 가중치 모형을 적용하여 산출한 값(0–1점)을 그대로 사용하였다.

#### 일반적 특성

일반적 특성으로는 성별, 연령, 교육 수준, 결혼 여부, 직업, 소득 수준을 포함하였다. 연령은 60세 이하, 70–79세, 80세 이상으로 구분하였다. 교육 수준은 초등학교 졸업 이하, 중학교 졸업 이상으로, 주거 형태는 독거, 비독거로, 거주지는 동, 읍•면으로 구분하였다.

#### 생활습관 관련 특성

생활습관은 음주 여부, 걷기운동 여부, 근력운동 여부, 하루 중 앉아있는 시간을 포함하였다. 음주는 비음주와 음주로 구분하였고, 걷기운동은 ‘주 5일 이상 한다’와 ‘하지 않는다’로, 근력운동은 ‘주 3일 이상 한다’와 ‘하지 않는다’로 구분하였다.

#### 건강관련 특성

건강관련 특성은 주관적 건강상태, 뇌질환 유병 여부, 심장질환 유병 여부, 골관절염 유병 여부, 고혈압 유병 여부, 스트레스 인지 정도, 체질량지수, 허리둘레, 당뇨병 유병기간, 혈당 조절 정도, 폐경기간을 포함하였다. 주관적 건강상태는 나쁨, 보통 이상으로 구분하였고, 스트레스 인지 정도는 원자료에서 ‘대단히 많이 느낀다’와 ‘많이 느낀다’는 ‘많이 느낀다’로 ‘조금 느끼는 편이다’와 ‘거의 느끼지 않는다’는 ‘조금 느낀다’로 재범주화 하였다. 비만도는 정상과 비만으로 구분하였고, 허리둘레는 대한비만학회 기준[14]에 따라 85 cm 미만은 정상, 85 cm 이상을 비만으로 분류하였다. 당뇨병 유병기간은 현재 나이에서 당뇨병 진단 연령을 뺀 값이다. 혈당 조절 정도는 혈당 조절 목표를 당화혈색소(hemoglobin A1c) 수치 6.5%와 7.0% 중 어느 기준을 선택할지에 대해 약간의 논란이 있다. 대한당뇨병학회[15]에서는 6.5% 미만을 권고하고 있고, 미국당뇨병협회[16]에서는 당뇨병 환자의 혈관 합병증을 줄이기 위해 7% 미만을 권고하였는데, 본 연구에서는 혈당 조절의 궁극적 목표를 당뇨병으로 인한 심혈관계 합병증 감소로 보고 당화혈색소 7.0%를 혈당 조절 기준으로 선정하였다. 이에 따라 혈당 조절군의 분류는 당화혈색소 7% 미만을 혈당 조절 양호군, 7%–8% 미만을 불충분군, 8% 이상을 불량군으로 분류하였다. 폐경기간은 현재 나이에서 폐경 연령을 뺀 값이다.

### 자료 분석

국민건강영양조사 자료는 대한민국에서 대표성 있는 표본을 추출한 확률표본이므로 복합표본분석을 시행하였다. 본 연구는 2016–2018년 자료로 3개년 자료의 통합 가중치를 사용하였고, 질병관리청에서 제공한 층화변수와 집락변수를 지정하여 분석하였다. IBM SPSS for Windows ver. 26.0 프로그램(IBM Corp., Armonk, NY, USA)을 사용하여 분석하였으며, *p*<.05일 때 통계적으로 유의하다고 판단하였다. 구체적인 분석방법은 다음과 같다.

1) 대상자의 건강관련 삶의 질 관련 요인(일반적 특성, 생활습관, 건강관련 특성)에 대해 서술적 통계분석을 하였다. 서술적 통계분석 결과는 가중치를 반영하지 않은 결과(n)와 가중치를 반영한 결과인 가중 퍼센트(weighted percent, W%), 평균과 표준오차로 제시하였다.

2) 건강관련 삶의 질에 대한 관련 요인은 complex samples general linear model (CSGLM) 단변량 분석을 이용하였다. 사후분석은 Bonferroni correction으로 검증하였다.

3) 건강관련 삶의 질에 대한 영향요인 분석은 CSGLM 다변량 분석을 이용하였다.

## Results

### 대상자의 일반적인 특성 및 생활습관에 따른 건강관련 삶의 질

본 연구에서 건강관련 삶의 질은 EQ-5D index로 계산하여 1점 만점에 평균 0.83±0.18점으로 매우 좋은 수준이었다. 본 연구대상자인 골다공증이 있는 폐경 후 당뇨 여성은 237명이었고, 연령은 70대가 43.3%로 가장 많았다. 교육 수준은 초등학교 졸업 이하가 75.1%, 주거 형태는 비독거가 67.9%, 동지역에서 사는 사람이 72.7%였고, 음주를 하는 사람은 30.1%로 나타났다. 주 5일 이상 걷기운동을 하는 여성은 34.6%, 주 3일 일상 근력운동을 하는 여성은 4.3%였고, 하루 중 앉아서 보내는 시간은 평균 9.10±0.29시간으로 나타났다(Table 1).

건강관련 삶의 질에 대한 복합표본 일반선형 분석에서 유의한 차이가 있었던 일반적 및 생활습관 특성은 연령, 교육 수준, 주 5일 이상 걷기운동, 주 3일 일상 근력운동, 하루 중 앉아서 보내는 시간이었다. 연령은 60대 이하(0.89점)가 삶의 질이 가장 높았고, 다음으로 70대(0.80점), 80대 이상(0.79점) 순이었으며(F=6.33, *p*=.002), 교육 수준은 중학교 졸업 이상(0.88점)이 초등학교 졸업 이하(0.82점)보다 높았다(t=2.20, *p*=.028). 주 5일 이상 걷기운동을 하는 군(0.89점)이 하지 않는 군(0.81점)보다 삶의 질이 유의하게 높았고(t=–3.04, *p*=.002), 주 3일 이상 근력운동을 하는 군(0.97점)이 하지 않는 군(0.83점)보다 삶의 질이 유의하게 높았으며(t=–6.43, *p*<.001), 하루 중 앉아서 보내는 시간이 많을수록 삶의 질은 유의하게 감소하는 것으로 나타났다(r=–0.33, *p*<.001) (Table 1).

### 대상자의 건강관련 특성에 따른 건강관련 삶의 질

건강관련 특성으로 주관적 건강상태가 나쁘다고 한 여성이 50.1%였고, 동반질환으로는 뇌질환이 9.1%, 심장질환은 10.6%, 골관절염은 46.7%, 고혈압은 97.8%였다. 스트레스를 많이 느낀다고 인지한 여성은 24.9%였고, 체질량지수가 비만에 속한 여성이 45.9%, 허리둘레가 비만에 속한 여성이 62.5%였다. 당뇨병 유병기간은 평균 11.37±0.83년이었고, 혈당 조절 정도가 양호한 사람은 58.4%, 불충분한 경우가 22.8%, 불량한 경우가 18.8%였다. 평균 폐경기간은 21.90±0.72년이었다(Table 2).

건강관련 삶의 질에 대한 복합표본 일반선형 분석에서 유의한 차이가 있었던 건강관련 특성은 주관적 건강상태, 골관절염 유병 여부, 스트레스 인지 정도, 당뇨 유병기간, 폐경기간이었다. 주관적 건강상태는 보통 이상(0.90점)이 나쁨(0.77점)보다 유의하게 삶의 질이 높았고(t=5.18, *p*<.001), 골관절염을 동반한 여성(0.79점)은 동반하지 않는 여성(0.88점)보다 낮았다(t=–3.34, *p*=.001). 스트레스를 조금 느낀다고 인지하는 여성(0.86점)이 많이 느끼는 여성(0.77점)보다 삶의 질이 높았고(t=–2.52, *p*=.012), 당뇨병 유병기간이 길수록 삶의 질은 유의하게 감소하는 것으로 나타났으며(r=–0.03, *p*<.001), 폐경기간이 길수록 삶의 질이 유의하게 증가하는 것으로 나타났다(r=–0.22, *p*<.001) (Table 2).

### 대상자의 건강관련 삶의 질에 영향을 미치는 요인

대상자의 건강관련 삶의 질에 영향을 미치는 요인을 파악하기 위해 복합표본 일반선형 분석을 시행하였다. 단변량 일반선형 분석에서 건강관련 삶의 질에 유의한 차이를 보인 변수와 선행 연구[5,6,8,9]에서 삶의 질과 연관이 있다고 밝혀진 요인인 연령, 교육 수준, 주거형태, 거주지, 음주경험, 주 5일 이상 걷기운동, 주관적 건강상태, 뇌질환, 심장질환, 골관절염, 고혈압 유병 여부, 스트레스 인지 정도, 체질량지수, 주 3일 일상 근력운동, 하루 중 앉아서 보내는 시간, 허리둘레, 당뇨병 유병기간, 폐경기간을 독립변수로 투입하였고, 종속변수는 건강관련 삶의 질을 투입하였다.

건강관련 삶의 질에 유의한 영향을 주는 변수는 연령, 주 5일 이상 걷기운동, 뇌질환 유무, 골관절염 유무, 고혈압 유무, 스트레스 인지 정도, 혈당 조절 정도, 허리둘레, 하루 중 앉아서 보내는 시간, 폐경기간이었다. 구체적으로는 연령이 70대가 60대 이하보다 삶의 질이 평균 0.15점 낮았고(t=–3.74, *p*<.001), 80대 이상은 60대 이하보다 평균 0.25점 낮았다(t=–3.42, *p*=.001). 주 5일 이상 걷기운동을 실천하지 않는 여성은 실천하는 여성보다 평균 0.11점 낮았다(t=–2.83, *p*=.005). 뇌질환이 있는 여성은 없는 여성보다 평균 0.32점 낮았고(t=–8.33, *p*<.001), 골관절염이 있는 여성은 없는 여성보다 평균 0.08점 낮았으며(t=–2.04, *p*=.044), 스트레스를 많이 느끼는 여성이 적게 느끼는 여성보다 삶의 질이 평균 0.06점 낮았다(t=–2.17, *p*=.032). 허리둘레는 비만한 여성이 정상인 여성보다 평균 0.14점 낮았고(t=–2.76, *p*=.007), 앉아있는 시간이 길수록 삶의 질이 유의하게 감소하는 것으로 나타났다(t=–2.10, *p*=.038). 이에 비해 고혈압이 있는 여성은 없는 여성보다 삶의 질이 평균 0.19점 높았고(t=2.03, *p*=.044), 혈당 조절 정도가 불량한 여성은 양호한 여성보다 평균 0.12점 높았으며(t=3.40, *p*=.001), 폐경기간이 길수록 삶의 질이 유의하게 증가하는 것으로 나타났다(t=3.09, *p*=.002) (Table 3).

## Discussion

본 연구의 종속변수인 건강관련 삶의 질은 개인의 삶에서 목표, 기대, 기준 및 관심사와 관련하여 자신의 위치에 대한 인식으로 정의하였으며[11] 평균 0.83점이었다. 이는 중년여성을 대상으로 동일한 도구인 EQ-5D로 측정한 선행연구의 0.93점[17]이나 골다공증을 가진 폐경 여성의 건강관련 삶의 질로 보고된 0.86점[11]보다 낮았다. 생애주기에 있어 폐경 후 여성은 노년기로 이행되면서 연령의 증가에 따른 신체적, 심리사회적 기능 저하와 함께 만성질환의 유병률이 높아져 삶의 질이 낮아지게 된다[11]. 즉, 본 연구대상자의 경우 골다공증과 당뇨병을 동반한 폐경 후부터 노년기에 이르는 여성으로 일반 중년여성이나 골다공증만을 가진 여성보다 건강관련 삶의 질이 더 낮았던 것으로 생각된다.

본 연구 대상자의 건강관련 삶의 질에 차이가 있는 일반적 특성 및 생활습관 관련 변수는 연령, 교육 수준, 주 5일 이상 걷기운동, 주 3일 이상 근력운동, 하루 중 앉아서 보내는 시간이었다. 연령이 높을수록 삶의 질이 낮았고, 교육 수준의 경우 초등학교 졸업 이하가 중학교 졸업 이상보다 삶의 질이 낮았는데, 이는 당뇨병 환자[5]나 폐경 후 골다공증 여성[6]에서 고연령군이나 낮은 교육 수준군에서 삶의 질이 더 낮다고 보고한 선행연구 결과를 지지하였다. 높은 교육 수준은 건강을 위한 지식이나 행태, 직업 등에 긍정적인 영향을 미쳐 삶의 질을 향상시키는 것으로 알려져 있어[18] 본 연구에서도 같은 맥락으로 해석할 수 있다.

생활습관은 신체기능이나 심리적인 건강의 유지•향상을 위해 매우 중요한 요인이다[19]. 본 연구에서는 건강관련 삶의 질이 주 5일 이상 규칙적인 걷기운동을 하거나 주 3일 이상 근력운동을 하는 여성이 운동을 하지 않는 여성보다 유의하게 높았고, 하루 중 앉아서 보내는 시간이 짧을수록 유의하게 높았다. 이는 여러 선행연구[19-22]에서 걷기운동 등의 유산소 운동과 근력운동을 규칙적으로 실천할 때 삶의 질이 높다는 결과[21], 활동적인 생활습관이 건강관련 삶의 질에 긍정적인 영향을 미친다는 결과[19]나 좌식시간이 길수록 삶의 질이 낮아진다는 결과[22]와 일치하였다. 폐경기는 복부지방의 축적이 가속화되는 시기로 운동을 통해 체지방의 제거뿐 아니라 인지기능 향상, 심혈관계 기능 증진 및 사회성을 증진시키는 효과가 있으므로[20] 만성질환을 동반한 중년기 이후 여성에서 연령과 신체기능에 적절한 신체활동을 지속적으로 실천하는 것이 매우 중요하며, 이를 위한 정부나 관련기관들의 적극적인 관심과 사회적 지지가 필요하다고 생각된다.

본 연구 결과 대상자의 건강관련 특성으로 주관적 건강상태, 골관절염 유무, 스트레스 인지 정도, 당뇨병 유병기간, 폐경기간에 따라 건강관련 삶의 질에 차이가 있었다. 주관적 건강상태는 자신의 현재 건강상태를 스스로 평가하는 것으로 사망률이나 만성질환의 강력한 예측 변인으로 작용한다[23]. 이는 현재의 건강상태를 신체적 측면뿐 아니라 사회적, 심리적인 측면에서 환자 자신의 주관적인 평가에 의해 얻을 수 있는 지표이기 때문에, 의학적인 진단과는 별도로 중요시하고 있다[23]. 본 연구에서는 주관적 건강상태 보통 이상이 나쁨보다 삶의 질이 유의하게 높았고, 골관절염을 동반하고 있는 경우 삶의 질이 더 낮았다. 당뇨병과 고혈압 환자에서도 주관적 건강상태가 좋을수록 삶의 질이 높게 나타났는데[24], 골관절염을 가지고 있는 경우 통증이나 신체기능의 제한, 스트레스의 증가 등으로 주관적 건강상태를 좋지 않게 인지하여 삶의 질을 낮게 지각할 수 있다[25]. 또한 골다공증과 함께 당뇨를 가지고 있는 본 연구 대상자의 경우 골관절염까지 동반하게 되면서, 통증과 함께 신체기능이 감소되고 일상생활 수행능력이 저하되어[26] 삶의 질이 더욱 낮아졌을 것이라 여겨진다. 따라서 폐경 후 다양한 만성질환을 가진 여성이라 할지라도 일상생활에서 신체활동을 최대한 유지하고, 저지방과 고비타민, 고단백 식이를 섭취하며, 취미생활 등을 통해 스트레스를 해소하는 등의 바람직한 생활방식을 개발하고 유지함으로써 건강에 관한 긍정적인 인지나 전반적인 만족감을 높일 수 있도록 돕는 것이 성공적 노후로 이어지는 중요한 요인이 될 것이라 생각한다.

본 연구 대상자의 건강관련 삶의 질에 영향을 미치는 요인들을 확인하기 위해 복합표본 다변량 선형 회귀분석을 시행한 결과에서 연령, 걷기운동 여부, 뇌혈관질환‧골관절염 고혈압 동반 여부, 스트레스 인지 정도, 혈당 조절 정도, 허리둘레, 하루 중 앉아서 지내는 시간, 폐경기간이 유의한 변수로 나타났다. 연령은 60대가 70대나 80대 이상보다 삶의 질이 더 높았다. 연령의 증가는 노화에 따른 건강 양상의 변화를 가져와 고혈압, 당뇨병 및 관절염 등과 같은 만성질환이 증가하게 되고[27], 여성은 폐경 이후 연령이 증가하면서 관절염이나 골다공증 등의 만성질환에 노출될 가능성이 현저히 증가하기 때문에 삶의 질이 떨어진다는 선행연구 결과[11]가 본 연구결과를 뒷받침하고 있다. 본 연구에서 뇌질환이나 골관절염을 동반한 경우에 그렇지 않은 경우보다 삶의 질이 낮았으나 고혈압을 가진 경우는 갖지 않은 경우보다 오히려 높은 것으로 나타났다. 이는 당뇨병 환자를 대상으로 한 Bang과 Hyeon [24]의 연구에서 고혈압을 가진 사람이 없는 사람보다 삶의 질이 더 낮았다는 결과와는 상반된다. 본 연구에서 고혈압을 가진 사람이 삶의 질이 높게 나타난 결과는 첫째, 본 연구의 대상자가 당뇨를 가진 폐경 후 여성으로 고혈압을 가진 사람이 97.8%로 대부분을 차지하여 단변량 분석에서는 유의미한 차이가 있다고 입증할 수 없었으나 다변량 분석에서는 다른 변수들과의 상호작용에 의해 유의한 차이를 나타낸 것으로 해석된다. 둘째로 삶의 질은 의학적 기준에서의 건강과 질병 수준을 의미하는 것이 아니라 주관적인 관점에서 평가한 것이므로[23] 고혈압으로 진단된 후에도 평소 뚜렷한 신체증상이나 불편감 등을 느끼지 못하여 삶의 질에 크게 영향을 미치지 않았던 것으로 해석된다. 그러나 고혈압으로 인한 불편감이나 증상을 느끼거나 합병증을 경험한 사람의 건강관련 삶의 질에 대한 추후 연구를 통해 본 연구결과를 재규명할 필요가 있겠다.

Lee 등[20]과 Lee [21]는 건강을 위한 운동을 꾸준히 할수록 삶의 질이 높다고 보고하였다. 본 연구에서도 걷기 운동을 주 5일 이상 하는 군이 하지 않는 군보다 삶의 질이 더 높았고, 이와 맥락을 같이하여 하루 중 앉아서 보내는 시간이 길수록, 그리고 허리둘레가 비만인 사람일수록 정상인 사람보다 삶의 질이 더 낮았다. 불충분한 신체활동은 비만도와 허리둘레를 증가시키고[28], 비만은 당뇨뿐 아니라 골다공증성 골절위험도를 증가시켜 건강관련 삶의 질을 낮추는 원인이 되는데[3], Oh 등[28]의 연구에서도 앉아있는 시간이 8시간보다 길어질수록, 그리고 허리둘레가 증가할수록 삶의 질이 낮아진다고 하여 본 연구 결과와 일치하였다. 세계보건기구에서는 비활동성 생활습관이 수명을 단축하고 다양한 질병을 일으킨다고 강조하였고, 좌식생활을 포함하는 불충분한 신체활동을 사망순위 10위 내에 포함되는 위험요인 중 하나로 발표하였다[22]. 걷기 운동은 대표적인 저‧중강도 운동으로 규칙적인 걷기만으로도 만성질환 유병률과 합병증을 감소시키는 효과가 있다[23]. 또한 걷기운동은 단순하지만 부상의 위험이 적어 모든 연령층에 적합하고 특히 당뇨 환자에 있어 지방조직의 인슐린 작용을 개선하여 혈당 조절에 도움이 되므로[15], 폐경 후 당뇨 여성에서 걷기를 실천하는 것은 삶의 질을 높이기 위해 매우 효과적이다. 또한 폐경기 여성의 건강을 유지하기 위해서는 유산소 운동과 함께 에너지 소비량과 근육량을 늘리는 근력운동이 필요하다[21]. 그러나 본 연구에서 주 3일 이상의 근력운동은 단변량 분석에서는 삶의 질과 관련이 있었으나 다변량 분석에서는 유의하지 않은 결과를 보였다. 이는 본 연구 대상자의 60%가 70대 이상 여성으로 근력운동을 하는 사람이 4.3%에 불과하여 정확한 결과 해석에 한계가 있다고 생각된다. 그러나 근력의 약화는 노화의 주 현상이며, 근력 강화운동은 골밀도를 유지하거나 증가시키고, 평형성이나 신체 활동력을 증진하므로[20] 개인의 체력이나 신체조건, 안전성 등을 고려한 유산소 및 근력운동을 지속적으로 수행할 수 있는 대책 마련이 시급하다고 생각된다.

당뇨병 환자에서 적극적이고 엄격한 혈당 조절은 당뇨병의 합병증 발생을 예방하거나 진행속도를 늦출 수 있는 가장 효과적인 방법으로 당화혈색소 수치가 1% 감소하면 미세혈관합병증은 37%, 심근경색은 14% 정도 감소된다[15]. 그러나 본 연구의 결과는 40세 이상의 당뇨병 환자의 40.2％만이 당화혈색소 7.0% 미만으로 조절되고 있으며, 혈당 조절이 불량한 군에서 당뇨 관련 스트레스가 높게 나타나 혈당 조절이 양호한 군에 비해 불량한 군이 삶의 질이 더 높게 나타났다고 보고한 연구결과[29]와 상반되었다. 당뇨병 환자의 관리는 청장년기에서는 합병증 예방이 목표이지만 노년기에서는 현재의 삶의 질을 먼저 중요시하는 경향이 있다[30]. 질병의 초기에는 질병의 위험성에 대한 두려움과 긴장감이 높아 치료 지시 및 자가관리 이행수준이 높으나, 유병기간이 길어질수록 혈당 조절이 불충분한 군에서는 혈당 조절 수준과는 관계없이 질병에 대한 민감성 감소와 함께 자가관리에 대한 부담감이나 스트레스를 덜 느끼게 되어[31] 오히려 삶의 질이 높았던 것으로 여겨진다. 그러나 당뇨병으로 인한 합병증 예방을 위해서 철저한 혈당 조절은 매우 중요하므로 자가관리나 치료 관련 스트레스가 높아져 혈당 조절이나 삶의 질에 부정적인 영향을 미치지 않도록 반복적인 혈당 측정과 식이제한, 합병증 관리에 따른 부담과 스트레스에 대한 관리 전략이 필요하다.

여성에 있어 폐경은 호르몬뿐 아니라 심리적, 신체적 변화로 인해 만성질환의 위험이 증가하고, 여러 가지 폐경 증상들과 더불어 삶의 질에 영향을 주게 된다[32]. 선행연구들에서는 폐경기간과 관련된 삶의 질에 대해 서로 상반된 결과들을 보고하고 있는데, 이는 폐경으로 인한 신체적 및 정서적 증상들은 일반적으로 폐경 이행기에 가장 심하고 시간이 지나면서 노인으로서의 삶에 적응을 하면서 점차 호전되는 것으로 설명하고 있다[31]. 본 연구에서는 단변량 분석에서는 폐경기간이 길어질수록 삶의 질이 낮은 것으로 나타났으나 다변량 분석에서는 폐경기간이 길어질수록 삶의 질이 높은 것으로 나타났다. Ashok 등[31]과 Choi와 Oh [32]는 폐경 초기보다 후기에 폐경에 대한 긍정적 태도를 보임으로써 삶의 질이 높다고 하여 본 연구와 일부 유사하였으나, Kim과 Ahn [6]의 연구에서는 폐경 후기 여성이 폐경 전기 여성 대비 삶의 질이 낮게 나타나 본 연구와 차이가 있었다. 폐경 여부와 폐경기간에 따른 삶의 질은 나이, 교육 수준, 소득 수준, 폐경 증상이나 건강 상태, 부부 친밀도, 폐경 이후의 불확실성 등 신체적, 심리•사회적 변수들과 관련이 있으며[33], 폐경기 동안의 삶의 질은 건강 상태와 생활습관 등 여러 변수들의 복잡한 상호작용에 의해 영향을 받는다[34]. 따라서 본 연구에서 단변량 분석과 다변량 분석 결과가 상이한 것은, 단변량 분석은 단순히 폐경기간과 삶의 질의 관계만을 고려하였지만 다변량 분석은 폐경 후 기간이 경과하는 동안 다양한 신체적, 사회적, 심리적인 요인들이 폐경기간과 교호작용을 일으키면서 폐경기간만을 반영한 결과와는 다른 방향으로 나타난 것으로 해석된다.

이상의 논의 결과 골다공증을 가진 폐경 후 여성의 당뇨병이 노년기로 이어져 중년기의 삶은 물론 노년기의 삶의 질까지 저해할 수 있으므로 골다공증과 당뇨병을 가진 폐경 후 여성의 삶의 질 향상을 위해 연령과 폐경기간 등 조절할 수 없는 요인을 제외하고 신체활동이나 생활습관의 개선, 스트레스 관리 등 변화 가능한 요인들에 초점을 두어 대상자의 폐경과 질병 관리능력을 향상시킬 수 있는 중재가 필요하겠다.

본 연구는 제7기 국민건강영양조사를 바탕으로 골다공증이 있는 폐경 후 당뇨 여성의 삶의 질 수준 및 영향요인을 한 시점에서 조사한 횡단적 조사연구로, 인과관계를 추론하는 데 신중을 기해야 한다. 또한 원자료에서 스트레스 인지를 한 개 문항으로 측정하여 스트레스를 충분히 반영하는 데 한계가 있었을 것으로 생각된다. 그리고 폐경 후 여성에서 교육 수준이나 주관적 건강상태가 삶의 질에 주요한 영향요인이라는 선행연구[17,23]와는 다르게 본 연구에서는 다변량 분석에서 유의한 영향요인으로 추출되지 않았다. 그럼에도 불구하고 우리나라 국민의 건강행태 및 건강수준을 대표하는 자료를 이용하여 고령화로 인해 골다공증과 당뇨병 유병률이 증가하고 있는 시점에서 이들 질환을 가진 폐경 후 여성의 삶의 질에 영향을 미치는 요인을 확인함으로써 폐경기에서 노년에 이르기까지 여성의 삶의 질을 높이기 위한 중재요인들을 제시하였다는 데 의의가 있다.

이상의 결과를 바탕으로 골다공증과 당뇨를 가진 폐경 후 여성에서 건강관련 삶의 질을 높이기 위해서는 지속적이고 효과적인 신체활동을 통해 당뇨병의 합병증을 예방하고 스트레스를 최소화하면서도 만성질환을 효과적으로 관리할 수 있도록 하는 중재전략을 적극적으로 시행할 필요가 있다. 추후 연구를 위한 제언으로는 중년기 이후 여러 만성질환을 함께 가지고 있는 여성뿐만 아니라 남성을 대상으로 만성질환 관리를 위한 자가관리나 약물 복용, 치료 지시 이행을 위한 스트레스 정도 및 이와 관련된 요인들을 파악하여 의료인들의 환자 관리 방안을 모색하는 시도가 필요하다.

## Figures and Tables

**Table 1.** Difference in health-related quality of life (HRQoL) according to general and lifestyle-related characteristics (N=237)

Characteristic	Categories	n (W%)^†^ or mean±SE^‡^	HRQoL						
			Mean±SE^‡^	t/F^§^/r	*p* ^∥^	B	SE	t	*p*
General characteristics									
Age (year)	≤69^a^	77 (39.9)	0.89±0.01	6.33	.002 (a>b>c)	Ref			
	70-79^b^	108 (43.3)	0.80±0.02			–0.09	.03	-3.09	.002
	≥80^c^	37 (16.7)	0.79±0.04			–0.10	.04	-2.38	.018
		71.12±7.21	0.83±0.18						
Education	≤ Elementary school	166 (75.1)	0.82±0.02	2.20	.028	Ref			
	≥ Middle school	56 (24.9)	0.88±0.02			0.06	.03	2.20	.028
Cohabitation	Alone	83 (32.1)	0.80±0.02	–1.83	.068	–0.05	.03	-1.83	.068
	Not alone	139 (67.9)	0.85±0.02			Ref			
Residence	Neighborhood	145 (72.7)	0.84±0.02	0.82	.411	0.02	.03	0.82	.411
	Town/township	77 (27.3)	0.82±0.02			Ref			
Lifestyle-related characteristics									
Drinking	Yes	38 (30.1)	0.81±0.03	–1.64	.101	–0.06	.03	-1.64	.101
	No	94 (69.9)	0.87±0.02			Ref			
Walking for exercise (more than 5 days a week)	Yes	74 (34.6)	0.89±0.02	–3.04	.002	Ref			
	No	148 (65.4)	0.81±0.02			–0.08	.03	-3.04	.002
Weight training (more than 3 days a week)	Yes	10 (4.3)	0.97±0.02	–6.43	<.001	Ref			
	No	212 (95.7)	0.83±0.01			–0.14	.02	-6.43	<.001
Weight training		9.10±0.29		-0.33	<.001	-0.02	.00	–4.02	<.001

Ref: reference.

† Unweighted count (weighted %);

‡ estimated mean±standard error;

§ Wald F;

∥ Bonferroni correction.

**Table 2.** Difference in health-related quality of life (HRQoL) according to health-related characteristics (N=237)

General characteristic	Categories	n (W%)^†^ or mean±SE^‡^	HRQoL						
			Mean±SE^‡^	t/F^§^/r	*p* ^∥^	B	SE	t	*p*
Health-related characteristics									
Subjective health status	Moderate or good	108 (49.9)	0.90±0.01	5.18	<.001	0.13	0.03	5.18	<.001
	Bad	114 (50.1)	0.77±0.02			Ref			
Cerebrovascular disease	Yes	16 (9.1)	0.79±0.06	–0.97	.331	–0.05	0.06	–0.97	.331
	No	206 (90.9)	0.84±0.01			Ref			
Heart disease	Yes	26 (10.6)	0.81±0.03	–0.87	.385	–0.03	0.04	–0.87	.385
	No	196 (89.4)	0.84±0.01			Ref			
Osteoarthritis	Yes	105 (46.7)	0.79±0.02	–3.34	.001	–0.08	0.02	–3.34	.001
	No	117 (53.3)	0.88±0.01			Ref			
Hypertension	Yes	153 (97.8)	0.82±0.02	–1.21	.227	–0.08	0.06	–1.21	.227
	No	3 (2.2)	0.89±0.06			Ref			
Perceived stress	A lot	53 (24.9)	0.77±0.03	–2.52	.012	–0.09	0.04	–2.52	.012
	A little	166 (75.1)	0.86±0.01			Ref			
Body mass index	Normal	124 (54.1)	0.83±0.02	0.02	.986	0.01	0.03	0.02	.986
	Obesity	94 (45.9)	0.83±0.02			Ref			
Waist circumference	Obesity	130 (62.5)	0.82±0.02	–1.47	.142	–0.04	0.03	–1.47	.142
	Normal	83 (37.5)	0.86±0.02			Ref			
Duration of DM (year)		11.37±0.83		–0.03	<.001	0.01	0.00	–0.37	.711
Glycemic control	Poor	38 (18.8)	0.86±0.04	0.43	.646	0.02	0.04	0.61	.545
	Insufficiency	48 (22.8)	0.82±0.03			–0.02	0.03	–0.54	.588
	Good	136 (58.4)	0.84±0.02			Ref			
Menopausal period (year)		21.90±0.72		–0.22	<.001	0.01	0.01	–2.53	.012

DM: Diabetes mellitus; Ref: reference.

† Unweighted count (weighted %);

‡ estimated mean±standard error;

§ Wald F;

∥ Bonferroni correction.

**Table 3.** Factors influencing health-related quality of life (HRQoL) (N=237)

Variable	HRQoL			
	B	SE	t	*p*
(Constant)	0.85	0.14	6.19	<.001
Age, ≥80 years^†^	–0.25	0.07	–3.42	.001
Age, 70–90 years^†^	–0.15	0.04	–3.74	<.001
Education, ≥ middle school^ †^	–0.05	0.04	–1.02	.311
Cohabitation, alone^†^	–0.01	0.03	–0.26	.796
Residence, city^†^	0.09	0.06	1.53	.129
Drinking, yes^†^	–0.01	0.03	–0.34	.734
Walking more than 5 days a week, no^†^	–0.11	0.04	–2.83	.005
Weight training more than 3 days a week, no^†^	–0.09	0.06	–1.61	.110
Sitting time per day	–0.01	0.00	–2.10	.038
Subjective health, moderate or good^†^	0.07	0.05	1.42	.157
Cerebrovascular disease, yes^ †^	–0.32	0.04	–8.33	<.001
Heart disease, yes^†^	0.06	0.07	0.91	.365
Osteoarthritis, yes^†^	–0.08	0.04	–2.04	.044
Hypertension, yes^†^	0.19	0.09	2.03	.044
Perceived stress, a lot^†^	–0.06	0.03	–2.17	.032
Body mass index, obesity^†^	–0.07	0.04	–1.65	.101
Glycemic control, poor^†^	0.12	0.03	3.40	.001
Glycemic control, insufficient^†^	0.01	0.04	0.32	.750
Waist circumference, obesity^†^	–0.14	0.05	–2.76	.007
Duration of diabetes mellites	0.01	0.01	1.02	.311
Menopausal period	0.01	0.01	3.09	.002
R^2^=.61, Wald F=10.23, *p*<.001				

† The reference groups were age (≤60 years), education (≤elementary school), cohabitant (non-living alone), residence (country), drinking (no), walking more than 5 days a week (yes), weight training more than 3 days a week (yes), subjective health (bad), cerebral disease (no), heart disease (no), osteoarthritis (no), hypertension (no), perceived tress (a little), body mass index (normal), glycemic control (good), and waist circumference (normal).
